# Regulation of *Arabidopsis* root development by small signaling peptides

**DOI:** 10.3389/fpls.2013.00352

**Published:** 2013-09-06

**Authors:** Christina Delay, Nijat Imin, Michael A. Djordjevic

**Affiliations:** Division of Plant Sciences, Research School of Biology, College of Medicine, Biology and Environment, The Australian National UniversityCanberra, ACT, Australia

**Keywords:** small signaling peptides, small post-translationally modified peptides, regulatory peptides, root development, CLE, RGF/CLEL/GLV, IDA, CEP

## Abstract

Plant root systems arise *de novo* from a single embryonic root. Complex and highly coordinated developmental networks are required to ensure the formation of lateral organs maximizes plant fitness. The *Arabidopsis* root is well-suited to dissection of regulatory and developmental networks due to its highly ordered, predictable structure. A myriad of regulatory signaling networks control the development of plant roots, from the classical hormones such as auxin and cytokinin to short-range positional signaling molecules that relay information between neighboring cells. Small signaling peptides are a growing class of regulatory molecules involved in many aspects of root development including meristem maintenance, the gravitropic response, lateral root development, and vascular formation. Here, recent findings on the roles of regulatory peptides in these aspects of root development are discussed.

## INTRODUCTION

The entire plant root system is formed post-embryonically from a single primary root. Growth and development of this system requires coordinated regulation of hardwired developmental programs together with input from environmental signals (Casimiro et al., 2003; Malamy, 2005). While the classical phytohormones are key players in many aspects of root development, cell-to-cell communication is a vital component of most developmental processes. Regulatory peptides are one class of small signaling molecules that mediate intercellular communication. Roles for small signaling peptides in shoot development have been elucidated and are relatively well-characterized (Fukuda and Higashiyama, 2011). Recently, there has been a leap in our understanding of the roles of regulatory peptides in root development.

Small signaling peptides arise from genes that typically encode an N-terminal signal peptide region, one or more conserved peptide domains and variable regions that flank one or both sides of the discrete peptide domains. There are two structural classes of signaling peptides: small (5–20 amino acids) post-translationally modified peptides, which are the focus of this review; and larger cysteine-rich peptides (approximately 50 amino acids) that undergo disulfide bond formation as part of the maturation process. Common post-translational modifications to the smaller peptides class include hydroxylation, sulfation, and arabinosylation. These modifications may increase peptide stability, assist with receptor interactions and provide a further degree of regulation. The precursor proteins undergo processing to form the mature peptide product. While the maturation process is poorly understood, it was recently shown that four residues upstream of the peptide domain are required for CLE (CLAVATA3/ESR-related) peptide endoproteolytic processing (Ni et al., 2011). Most peptides are thought to act as extracellular signaling molecules that are ligands for membrane bound receptors although few ligand/receptor interactions have been validated (Matsubayashi et al., 2002; Hirakawa et al., 2008; Ogawa et al., 2008).

Several families of regulatory peptides, defined by homology of the peptide domain, have been implicated in various developmental processes in the roots. The CLE peptide family has a conserved 12–14 amino acid CLE motif at or near the C-terminus (Cock and McCormick, 2001). The RGF (ROOT GROWTH FACTOR) family of peptides, also known as GLV (GOLVEN) and CLEL (CLE-Like), has a conserved 14 amino acid peptide domain containing the tyrosine sulfation motif Asp-Tyr (Matsuzaki et al., 2010; Meng et al., 2012b; Whitford et al., 2012). The IDA (INFLORESCENCE DEFICIENT IN ABCISSION) peptide family has conserved, functionally active 20 amino acid motif (EPIP; Butenko et al., 2003; Stenvik et al., 2008). The CEP (C-TERMINALLY ENCODED PEPTIDE) family has a conserved 15 amino acid peptide domain with two proline residues that may be hydroxylated (Ohyama et al., 2008).

**FIGURE 1:**
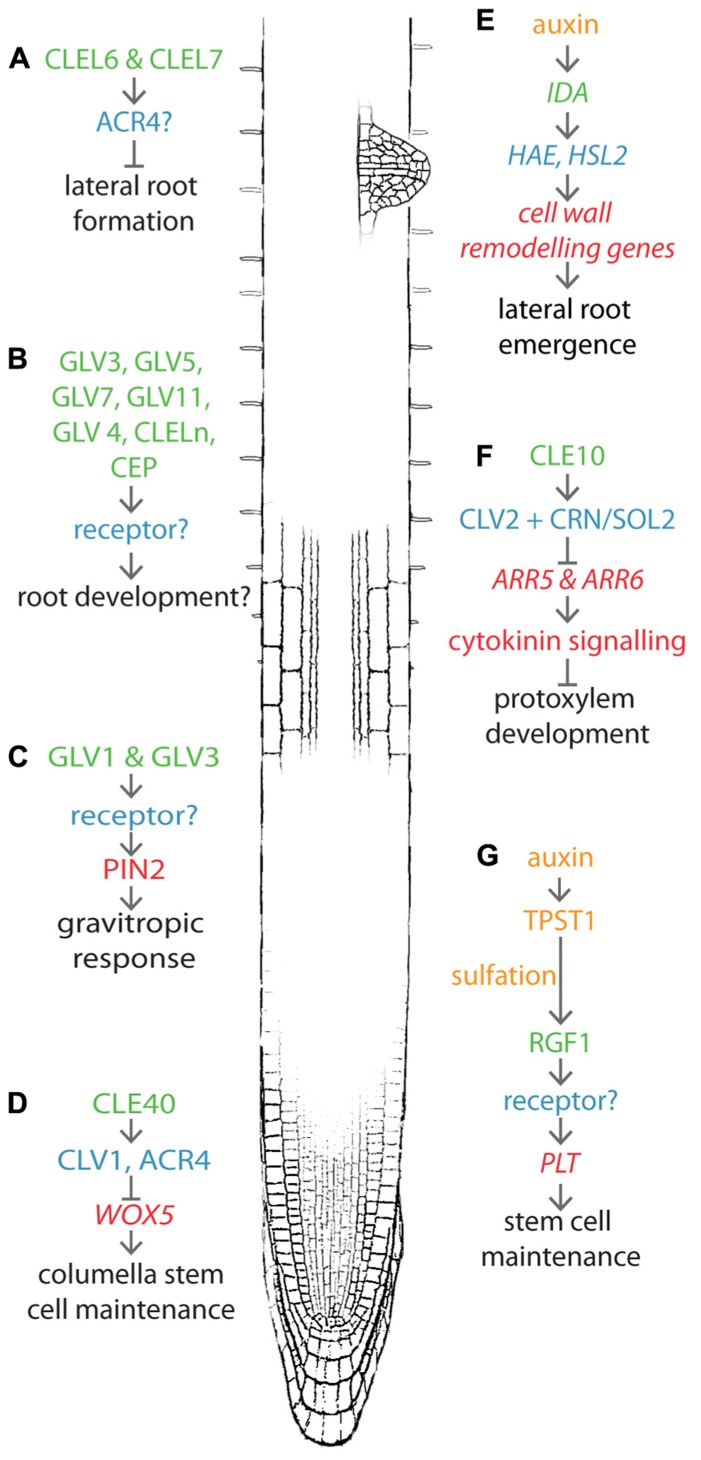
**Recent advances in peptide-mediated regulation of root development.** CLE, RGF/GLV/CLEL, IDA, and CEP peptides are involved in several aspects of root development including lateral root formation **(A), (B), (D), (E)**, protoxylem development **(F)**, stem cell maintenance **(G)**, and gravitropic response **(C)**. Known pathways discussed in this review are shown near their approximate location in the root. Peptides are indicated in green text, receptors in blue. Upstream processes are yellow and downstream processes are red. Developmental output is indicated by black text.

Recent work implicates regulatory peptides in many aspects of root growth and development, including meristem maintenance, gravitopism, lateral root (LR) development, and protoxylem differentiation (**Figures 1A–G**). This review outlines the recently discovered roles of these peptide families in root development.

## ROOT MERISTEM MAINTENANCE

For continual growth and development, roots require a source of new cells that are able to differentiate into various tissue types. The root meristem contains a population of stem cells maintained by the quiescent center (QC). An extraordinary amount of precise regulation is required to ensure that the identity of the QC cells, the surrounding stem cells and their daughters is maintained. Several transcription factors are essential for this. *WOX5* (*WUSCHEL*-related homeobox 5) is expressed in the QC to maintain stem cell identity (Haecker et al., 2004). *SCR* (*SCARECROW*) and *SHR* (*SHORTROOT*) are expressed along the radial axis to provide positional information and also maintain QC identity (Di Laurenzio et al., 1996; Helariutta et al., 2000; Sabatini et al., 2003). *PLT1* (*PLETHORA1*) and *PLT2* (*PLETHORA2*) are auxin responsive genes that form a gradient distribution to maintain stem cell identity when expressed at high levels and promote differentiation when present at low levels (Aida et al., 2004; Galinha et al., 2007). An auxin maximum is also required in the stem cell niche (Sabatini et al., 1999; Friml et al., 2003).

A recent report implicated the CLE40 peptide in maintaining QC and columella stem cell identity (**Figure 1D**; Stahl et al., 2009). *CLE40* is expressed in the stele and in differentiating columella cells. *cle40* loss of function mutants had short roots with irregular tips caused by delayed differentiation of columella stem cells. Excessive CLE40 led to columella stem cell differentiation. The expression of *WOX5* was perturbed when CLE40 was deregulated, extending beyond the QC in the *cle40* mutant and being reduced in the QC and shifted toward the proximal meristem upon CLE40 peptide addition to WT plants. CLE40, together with ABR4 (*Arabidopsis *CRINKLY 4), a receptor-like kinase, regulated the spatial expression of *WOX5* to maintain columella stem cell identity. Additionally, the CLV2 (CLAVATA 2) leucine-rich repeat receptor was found to be necessary for CLE40-dependent root growth suppression, but not for differentiation of the daughters of columella stem cells (Stahl et al., 2009), indicating that the same peptide may interact with more than one receptor to regulate different developmental processes. Indeed, further investigation showed that subcellular localization, environment, and concentration affect the formation of receptor complexes (Stahl et al., 2013). The leucine-rich repeat receptor kinase CLV1 was shown to be involved in the signaling pathway of CLE40 and ABR4 (**Figure 1D**). *ABR4* and *CLV1* have similar expression patterns in the root meristem. CLE40 regulates *ABR4* expression but not *CLV1* expression. Using Förster resonance energy transfer and multiparameter fluorescence image spectroscopy, the transmembrane domains of ABR4 and CLV1 were shown to interact and form homomeric and heteromeric complexes depending on localization (plasma membrane or plasmodesmata). Both the *clv1* and *aBR4* single mutants were impaired in aspects of meristem maintenance and responded differently to CLE40 peptide application. This indicated that the homo- and heteromeric complexes differentially regulate root meristem maintenance.

In another recent report, the sulfated RGF1 peptide was found to regulate meristem maintenance by providing a link between *PLT* expression and auxin through sulfation by TPST1 (TYROSYLPROTEIN SULFOTRANSFERASE 1; **Figure 1G**; Matsuzaki et al., 2010; Zhou et al., 2010). The *tpst1* mutant displayed pleiotropic effects including decreased meristem size and cell division activity, QC misregulation and starch granule accumulation. This mutant lacked the only copy of tyrosylprotein sulfotransferase found in the *Arabidopsis* genome (Matsuzaki et al., 2010; Zhou et al., 2010). *TPST1* was induced by auxin and helped to maintain auxin distribution by regulating expression of *PIN*-*FORMED (PIN)* and auxin biosynthetic genes (Zhou et al., 2010).

The RGF peptide family was found by a bioinformatic search for putatively sulfated peptide-encoding genes (Matsuzaki et al., 2010). Applying synthetic, sulfated RGF1 peptide to *tpst1* roots ameliorated the stem cell misregulation phenotype and increased cell division activity, indicating that sulfated RGF1 was required for these processes. Further investigation showed the sulfated peptides PSK (PHYTOSULFOKINE) and PSY1 (PLANT PEPTIDE CONTAINING SULFATED TYROSINE 1) were also required for full restoration of cell division activity. Eight of the nine sulfated RGF peptides complemented the *tpst1* mutant in this way, however, non-sulfated peptides were unable to do so. It was found that RGF1 acts by regulating spatial expression and expression levels of *PLT* transcription factors. This work provided a link between auxin levels, the RGF peptide and *PLT* expression through TPST1. These studies also exemplified the extra layer of regulation that can be provided by post-translational modification of signaling peptides.

The *CEP* family has also been shown to affect root meristem development (Ohyama et al., 2008). Several forms of cleaved and modified peptides were confirmed using mass spectrometry on *CEP1* overexpression lines. Synthetic 15 amino acid CEP1 peptide application or *CEP1* overexpression arrested root growth. Meristematic cell division and expansion was repressed by CEP1, although QC specification was not affected. A more detailed analysis of the CEP family and the mode of action of individual CEPs is required.

## VASCULAR DEVELOPMENT

Once cells leave the meristematic zone of the root, they go through phases of elongation and differentiation into specific tissue types. Procambial and cambial cells differentiate into xylem and phloem, the vascular tissues required for long-distance transport of water and nutrients though the plant. Several regulatory peptides are known to play important roles in vascular development, including the CLE-family member TDIF (TRACHEARY ELEMENT DIFFERENTIATION INHIBITORY FACTOR; Ito et al., 2006). More recently, another member of this family, CLE10, was found to inhibit protoxylem formation through interaction with cytokinin signaling (**Figure 1F**; Kondo et al., 2011).

A screen for *CLE* genes up-regulated during vascular development together with synthetic peptide assays implicated *CLE10* in early xylem differentiation (Kondo et al., 2011). CLE10 peptide application rapidly down-regulated the transcription of *ARR5* and *ARR6*, two type A *Arabidopsis* *RESPONSE* *REGULATORs* that negatively regulate cytokinin signaling. This indicated that cytokinin signaling was increased by CLE10, leading to decreased protoxylem development, one of the documented roles of cytokinin. The leucine-rich repeat receptor-like protein CLV2 and protein kinase CRN (CORYN)/SOL2 (SUPRESSOR OF LLP2) were both required for CLE10 suppression of *ARR5* and *ARR6* transcription, indicating they may be the receptor module for the CLE10 peptide.

## GRAVITROPIC RESPONSE

Response to gravity stimulus is an essential component of root development. This process relies on gravity sensing in the root tip together with manipulation of auxin gradients, mediated by the localization of the *AUX1*/*LIKE*-*AUX1* family of auxin influx carriers and the *PIN* family of auxin efflux carriers (Vieten et al., 2007). The GLV/RGF peptide family has been implicated in this process (**Figure 1C**). Using a reverse genetic screen, Whitford et al. (2012) found that *GLV1/RGF6*, *GLV2/RGF9*, and *GLV3/RGF4* overexpression (or sulfated peptide addition) caused an altered gravitropic response in roots. GLV1/RGF6 and GLV3/RGF4 post-transcriptionally regulated PIN2 within minutes of peptide application, presenting evidence that GLV/RGF signaling controls the trafficking and stability of PIN2. A perturbation in the PIN2 pool would abolish the finely tuned auxin gradient necessary for normal gravitropic response. A loss of function *glv3*/*rgf4* mutant displayed gravitropic defects (Fernandez et al., 2013) implying GLV3/RGF4 is required for correct gravitropic response.

In a separate study, *CLEL*/*RGF* genes were found by homology with *CLE18*, a peptide precursor gene with a CLE motif in the variable region and a CLEL/RGF peptide domain at the C-terminus (Meng et al., 2012a). Unmodified CLEL6/RGF6 and CLEL8/RGF1 peptide application and *CLEL6/RGF6* and *CLEL7/RGF5* overexpression induced long roots on WT plants as well as an additional “wavy” root phenotype. This wavy root phenotype was reported to be independent of thigmotropism and phototropism. It was also reported to be independent of gravitopism as the phenotype persisted when unmodified CLEL6/RGF6 peptide was applied to *eir1-1 *and * aux1-7 *mutants, which are impaired in the gravitropic response. This is in disagreement with the aforementioned study which implicates CLEL6/GLV1/RGF6 in gravitropic response.

Interestingly, the peptide-encoding gene at the center of this debate, *CLEL6*/*GLV1/RGF6*, is reportedly not expressed in any part of the root (Whitford et al., 2012; Fernandez et al., 2013). Furthermore, a *GLV1*/*RGF6* knockdown line did not show impaired gravitropic response (Fernandez et al., 2013). This indicates regulation of peptide gene expression is paramount to its *in planta* function. Excessive peptide treatment by overexpression or peptide application should be interpreted in a biologically relevant context and should be supported by data from loss of function mutants.

## LATERAL ROOT DEVELOPMENT

The formation of LRs allows the plant to exploit water and nutrients in the surrounding soil. LRs arise from a repetitive process which begins in the root meristem region. The earliest described event in LR development is priming or pre-branch site formation. This process requires auxin (De Smet et al., 2007) and the oscillation of over 3,000 genes (Moreno-Risueno et al., 2010). As the cells move into the differentiation zone, a site of relatively low auxin levels, LR founder cells are specified from xylem pole pericycle cells (Dubrovsky et al., 2008, 2011). The founder cells are initiated in an auxin-dependent manner (Dubrovsky et al., 2011) and begin to undergo a series of divisions. Eight stages of LR development have been defined (Malamy and Benfey, 1997), from the first asymmetric division (stage I) through many subsequent rounds of cell division (stages II–VII), to LR emergence (stage VIII). In order for the nascent LR to pass through the cortical, endodermal, and epidermal cell layers before emerging, auxin-dependent degradation and remodeling of cell walls is required (Swarup et al., 2008).

Peptides from the RGF family were reported to regulate LR development in an auxin-independent manner (**Figure 1A**; Meng et al., 2012a). Overexpression of *CLEL6* /*RGF6* or *CLEL7/RGF5* resulted in a significant reduction in LR number, due to abnormal cell divisions at stage I of LR development. The authors speculated that CLEL6/RGF6 and CLEL7/RGF5 may interact with the receptor kinase ABR4, as it too plays an important role in the early stages on LR development. Other RGF genes were found to be specifically expressed during different stages of LR development (Fernandez et al., 2013). Promoter reporter constructs showed that *GLV6*/*RGF8* was active from stage I of LR development, *GLV5/RGF2* and *GLV10/RGF5* at stage II, *GLV7/RGF3* and *GLV11/RGF1* at stage IV, *GLV3/RGF4* at stage V, and *GLV9* and *GLV2/RGF9* after emergence. Overexpression of these genes resulted in decreased LR number as well as root waving and enlarged root meristems. These data indicate that RGF peptides may act at different stages of LR development, however, specific mechanisms remain to be elucidated.

The IDA peptide and its receptors, HAE (HAESA) and HSL2 (HAESA-Like 2) were recently shown to play a role in LR emergence (**Figure 1E**; Kumpf et al., 2013) in addition to their known roles in floral abscission (Butenko et al., 2003; Stenvik et al., 2008). The *ida*, * hae*, * hsl2 *single mutants, and *hae hsl2 *double mutant showed a significant reduction in the number of LRs (Kumpf et al., 2013). LR primordia in these mutants encountered difficulties in penetrating the cortical, endodermis, and epidermal cell layers and often displayed irregular flattened shapes. *IDA* was strongly induced by auxin, whereas the two receptors were only transiently induced by auxin, indicating the receptors are used to limit IDA function. Two stages for IDA-mediated cell wall remodeling (CWR) were identified. During early primordia development (stage I and II), auxin from the LR primordium induced *IDA* expression in the endodermal cells, where HAE and HSL2 were already present. IDA signaling led to the expression of CWR enzymes, which allowed the nascent LR to pass through the endodermal cell layer. At a later stage in primordia development (stage V), auxin was derived from the auxin influx carrier LIKE AUX1-3 (LAX3), expressed in the neighboring cortical and epidermal cells. This induced the degradation of SOLITARY ROOT1 which in turn released the transcription factors AUXIN RESPONSE FACTOR (ARF) 7 and 19. ARF7 was required for the subsequent induction of *IDA* that triggered the expression of CWR genes through HAE/HSL2 signaling to allow the primordia to emerge from the parent root.

## OTHER ROLES OF SMALL SIGNALING PEPTIDES IN ROOT DEVELOPMENT

A recent report described *CLELn *(*CLE*-Like protein in the nucleus), a RGF family gene that lacked the archetypal N-terminal signal sequence (Meng et al., 2012b). A green fluorescent protein (GFP)-fusion assay suggested this peptide localizes to the nucleus and western blots indicated it is specifically processed from the precursor. Overexpression of this gene gave a long root phenotype similar to *GLV2/RGF9* overexpression, however, synthetic peptide application elicited the long and wavy phenotype seen when GLV1/RGF6 and GLV11/RGF1 synthetic peptides were assayed. These data raise the possibility that regulatory peptides do not act solely as intercellular signals and may play roles in nuclear signaling or are secreted by non-conventional routes.

Regulatory peptides also play important roles in nodule organogenesis in legumes. Nodules form in response to infection by symbiotic bacteria called rhizobia. This process requires systemic regulation (autoregulation of nodulation; Caetano-Anolles and Gresshoff, 1991), a process that has parallels with shoot meristem regulation in *Arabidopsis* by *CLV3* and *CLV1 *and their downstream processes (Mayer et al., 1998; Brand et al., 2000; Schoof et al., 2000; Leibfried et al., 2005; Osipova et al., 2012). Two root-specific pathways that involve Nod factors and cytokinin signaling are also required for nodulation. *Medicago truncatula CLE12 *and* CLE13 *are up-regulated in nodulating roots (Mortier et al., 2010). Upon overexpression, wild type plants do not form nodules. Suppression of nodulation by *Mt*CLE12 and *Mt*CLE13 is dependent upon *SUNN* (*SUPER* *NUMERIC* *NODULES*, orthologous to *CLAVATA1* in *Arabidopsis*) and induces type A response regulators, leading to cytokinin signaling (Mortier et al., 2010; Saur et al., 2011). Similar results were found in *Glycine max* (Reid et al., 2011) and *Lotus* *japonicus* (Okamoto et al., 2009), indicating at least two CLE peptides play an essential role in the autoregulation of nodulation. Recently a nodule-specific CLE in *L. japonicas* (CLE-RS2) was shown to be a root produced arabinosylated peptide (Okamoto et al., 2013). It interacted with HAR1, which shares functional similarity with SUNN, in an arabinose chain and sequence-dependent manner. CLE-RS2 was found in shoot-collected xylem sap, indicating that the CLE-RS glycopeptide is a long distance, root-to-shoot signal that controls autoregulation. These exciting results add a new dimension to how regulatory peptides control root development.

## FUTURE PERSPECTIVES

Although a number of peptides from the CLE and RGF families have been implicated in different aspects of root development, the specific function and mechanistic action of other family members remains to be elucidated (**Figure 1B**). In a step toward this, the expression patterns and effects of misregulation of all RGF and CLE peptide family members have been assessed (Jun et al., 2010; Fernandez et al., 2013). Further work is required to elucidate the functions of the CEP family in root development.

While genetic redundancy has hindered elucidation of peptide function by loss of function mutants, a recently reported technology may assist in overcoming this. It was shown that by substituting Gly6 in the *CLV3* peptide domain for Ala or Thr, dominant-negative *clv3 *phenotypes were obtained (Song et al., 2013). When applied in combination with the unsubstituted CLV3 peptide, it was shown that the antagonistic effect was a result of competition between the two peptides. It was hypothesized that CLV3_Thr6_ was able to bind the CLV3 receptor without eliciting a response. This concept was also used to make antagonists for CLE8 and CLE22 peptides. This technology may assist in elucidating the function of specific regulatory peptides.

As highlighted in this review, small signaling peptides play important roles in root development. There are several layers of regulation which serve to add specificity to the roles of individual peptides. These include post-translational modification, tissue-specific expression, regulation of receptor expression, the subcellular localization of the receptor and the potential for long-distance movement. Recent bioinformatic approaches have indicated that over 7,000 small, unannotated open reading frames exist in the *A.* *thaliana *genome (Hanada et al., 2013). As proportion of these are likely to be regulatory peptide-encoding genes, there is still a long way to go in fully exploring the extent of peptide-mediated developmental regulation.

## Conflict of Interest Statement

The authors declare that the research was conducted in the absence of any commercial or financial relationships that could be construed as a potential conflict of interest.

## CONTRIBUTION

Christina Delay wrote the paper. Nijat Imin and Michael A. Djordjevic critically read and edited the paper.
